# *In vitro* propagation and analysis of secondary metabolites in *Glossogyne tenuifolia* (Hsiang-Ju) - a medicinal plant native to Taiwan

**DOI:** 10.1186/s40529-014-0045-7

**Published:** 2014-06-24

**Authors:** Chia-Chen Chen, Hung-Chi Chang, Chao-Lin Kuo, Dinesh Chandra Agrawal, Chi-Rei Wu, Hsin-Sheng Tsay

**Affiliations:** 1 Department of Chinese Pharmaceutical Sciences and Chinese Medicine Resources, China Medical University, Taichung, Taiwan; 2 Department of Golden-Ager Industry Management, Chaoyang University of Technology, Taichung, Taiwan; 3 Department of Applied Chemistry, Chaoyang University of Technology, No.168, Gifong E. Rd., Taichung, 41349 Wufong Taiwan

**Keywords:** Glossogyne tenuifolia, In vitro plant regeneration, Medicinal plant, Micropropagation, Luteolin, Oleanolic acid, Shoot tip culture

## Abstract

**Background:**

*Glossogyne tenuifolia* Cassini (Hsiang-Ju in Chinese) is a perennial herb native to Penghu Islands, Taiwan. The herb is a traditional anti-pyretic and hepatoprotective used in Chinese medicine. Several studies on *G. tenuifolia* have demonstrated its pharmacological values of antioxidation, anti-inflammation, immunomodulation, and cytotoxicity on several human cancer cell lines. Active compounds, oleanolic acid and luteolin in *G. tenuifolia* are affected by several factors, including climatic change, pathogens and agricultural practices. Plant population of *G. tenuifolia* has been severely affected and reduced considerably in natural habitat due to the use of herbicides by farmers. Also, collection of plant material from the natural habitat is restricted to a few months in a year. Therefore, the objective of the present study was to develop an efficient micropropagation protocol for *G. tenuifolia.* The study also aimed to investigate the influence of *in vitro* growth environment on the active compounds in *in vitro* shoots, tissue culture raised greenhouse plants; compare the values with wild plants and commercially available crude drug.

**Results:**

Half-strength MS (Murashige and Skoog) basal medium supplemented with 0.1 mg/L 6-benzyladenine (BA) and 0.1 mg/L α-naphthaleneacetic acid (NAA) induced the maximum average number of shoots (7.3) per shoot tip explant excised from *in vitro* grown seedlings. Induction of rooting in cent percent *in vitro* shoots with an average number of 6.6 roots/shoot was achieved on ½ strength MS medium supplemented with 3.0 mg/L indole-3-acetic acid (IAA). The rooted plantlets acclimatized successfully in the greenhouse with a 100% survival rate. HPLC analysis revealed that the quantity of oleanolic acid and luteolin in *in vitro* shoots, tissue culture plants in the greenhouse, wild type plants and commercial crude drug varied depending upon the source. The oleanolic acid and luteolin contents were found to be significantly higher (16.89 mg/g and 0.84 mg/g, respectively) in 3-month old tissue culture raised plants in greenhouse compared to commercially available crude drug (6.51 mg/g, 0.13 mg/g, respectively).

**Conclusions:**

We have successfully developed an *in vitro* propagation protocol for *G. tenuifolia* which can expedite its plant production throughout the year. The contents of oleanolic acid and luteolin in the tissue culture raised plants in the greenhouse were significantly higher than the marketed crude drug demonstrating the practical application of the tissue culture technology. These findings may be very useful in micropropagation, germplasm conservation and commercial cultivation of *G. tenuifolia*. So far, there is no published report on tissue culture propagation of this important medicinal plant species.

**Electronic supplementary material:**

The online version of this article (doi:10.1186/s40529-014-0045-7) contains supplementary material, which is available to authorized users.

## Background

Medicinal herbs have played a significant role in maintaining human health and improving the quality of life for thousands of years. Many active phytochemicals, including flavonoids, terpenoids, lignans, sulfides, polyphenolics, carotenoids, coumarins, saponins, plant sterols, curcumins, and phthalides, have been identified (Craig [1999]). Some of these phytochemicals have been found to be potent antioxidants, metal chelators, or free radical scavengers, which may account for their health promoting properties (Cotell et al. [1996]). Today, medicinal plants are important to the global economy as approximately 85% of traditional medicine preparations involve the use of plants or plant extracts (Vieira and Skorupa [1993]). In the past few decades, there has been a resurging interest in the study and use of medicinal plants in health care and in recognition of the importance of medicinal plants to the health system (Hoareau and DaSilva [1999]). This has led to an exponential rise in demand for herbal medicines, and also a considerable international awareness about the dwindling supply of the world’s medicinal plants. Therefore, all possible modes of plant propagation and large scale cultivation have been explored. In our laboratory, tissue culture techniques have been used successfully for propagation of several medicinally important plant species (Tsay [1999]; Nalawade et al. [2003]; Mulabagal and Tsay [2004]; Tsay and Agrawal [2005]; Chen et al. [2006] and Chang et al. [2007]). Plants propagated by tissue culture have been reported to show less variation in the content of secondary metabolites than their cultivated or wild counterparts (Yamada et al. [1991]).

*G. tenuifolia* Cassini belonging to the family Asteraceae originates from Penghu Islands, Taiwan (Li [1978]). The perennial herb has been used to make traditional healthy food and herbal tea on the island for a long time. It is a traditional anti-pyretic and hepatoprotective used in the Chinese system of medicine (Anonymous [1999]). Plant decoction has been used for treatment of lung diseases, chronic nephritis, edema and prevention of sunstroke (Xu [1972]). The main active compounds of *G. tenuifolia* are luteolin, luteolin-7-glucoside and oleanolic acid (Anonymous [1999]). Several studies on *G. tenuifolia* have demonstrated its pharmacological values of antioxidation (Wu et al. [2005a]; Yang et al. [2006]), anti-inflammation (Wu et al. [2004]; Hsu et al. [2005]); immunomodulation (Ha et al. [2006]) and cytotoxicity on several human cancer cell lines (Hsu et al. [2005]). The ethanol extracts of *G. tenuifolia* showed strong ROS scavenging (antioxidant) activity in both cell free and cell based systems (Wu et al. [2005b]). Also, glossogin, an effective component of *G. tenuifolia* has been found to be a promising chemotherapeutic agent against lung cancer (Hsu et al. [2008]). It was demonstrated that the bioactive fraction ‘Fr. C’ of *G. tenuifolia* significantly inhibited the proliferation of A549 lung cancer cells (Hsu et al. [2011]). *G. tenuifolia* extract reportedly reduces the synthesis of the inflammatory mediator in activated murine macrophages RAW264.7 via an NF-κB-dependent pathway (Wu et al. [2004]). *G. tenuifolia* extract has also been found to inhibit the synthesis of a pro-inflammatory mediator in activated murine peritoneal macrophages, partially via NF-jB-dependent pathways (Ha et al. [2006]). In another study, *G. tenuifolia* extract suppressed the survival of mature osteoclasts by inhibiting osteoclast survival signaling pathways including NF-B, JNK, p38 and Akt, indicating its potential in the osteoclast-related diseases such as osteoporosis (Wang et al. [2014]).

Flavor of ‘Xiang-Ru tea’ (made from *G. tenuifolia* ) is very popular in Taiwan. The Kaohsiung District Agricultural Improvement Station has developed canned ‘Xiang-Ru tea’ and ‘Xiang-Ru jelly’, and Penghu County farmers have come out with ‘Xiang-Ru tea’ bags. Additionally, other *G. tenuifolia* products and technologies are being developed, indicating an immense economic potential of the herb. However, the supply of natural plant material is seasonal and restricted to only a few months. Due to these constraints, production of *G. tenuifolia* plants having higher levels of active compounds and sustainability of its supply throughout the year are important issues. The development of a micropropagation protocol of *G. tenuifolia* offers the potential to alleviate these problems.

The purpose of this study was to develop a protocol for *in vitro* propagation of *G. tenuifolia.* The study also aimed to estimate the quantities of luteolin and oleanolic acid compounds in tissue culture raised plants; compare them with the quantities found in wild types and commercial crude drug using High-performance liquid chromatography (HPLC). The method developed in the present study can be applied for the mass production of true-to-type *G. tenuifolia* plants of superior genotypes at a commercial scale.

## Methods

### Establishment of aseptic cultures

Seeds of *G. tenuifolia* were collected from Penghu Islands, Taiwan. Wild type plant materials were collected from Chimei and Wangan islands, Taiwan. Samples of commercial drug of *G. tenuifolia* were obtained from the authorized source. Seeds were surface disinfected by washing several times with sterile distilled water, followed by dipping in 70% (v/v) ethanol for 10s, then immersing in a solution of 1% (v/v) sodium hypochlorite containing 1 drop of Tween-20 for 5 min. Final washing consisted of 3 rinses of 5 min each in sterile distilled water. Thereafter, to raise *in vitro* seedlings, the disinfected seeds were cultured on ½X (half), and 1X (full) strength of macro, micro nutrients and vitamins of Murshige and Skoog’s (Murashige and Skoog [1962]) medium, hereinafter referred as MS basal medium. The pH of the culture media was adjusted to 5.7 ± 0.1 before autoclaving. All media were gelled with 0.9% Difco Bacto-agar (Difco Laboratories, Detroit, MI, USA).

### Multiple shoots induction

For induction of multiple shoots in *G. tenuifolia* , initial experiments were performed to find out a suitable culture medium, hence four different basal media, i.e. MS (Murashige and Skoog [1962]); WPM - Woody plant medium (Lloyd and McCown [1981]); B5 (Gamborg et al. [1968]); and N6 (Chu et al. [1975]); different concentrations of plant growth regulators (PGRs) e.g. BA (6-benzyladenine), Kin (Kinetin) and NAA (α-naphthaleneacetic acid); and different concentrations of sucrose were tested in a randomized design. These trials gave us some idea of the growth regulators and the best basal medium needed to induce shoot induction in *G. tenuifolia* . Thereafter, shoot tips (0.8 to 1 cm long) were excised from 5 weeks old *in vitro* raised seedlings and were cultured on MS basal medium supplemented with 0.1, 0.5, 1.0 mg/L of 6-benzyladenine (BA) or 0.1, 0.5, 1.0 mg/L of kinetin and a fixed concentration (0.1 mg/L) of α-naphthaleneacetic acid (NAA), 3% sucrose and 0.9% agar. The pH of all media was adjusted to 5.7 ± 0.1. Glass bottle (650 ml capacity), each having 100 ml of medium was used as culture vessel. After inoculation, the cultures were incubated in a growth room at 25 ±°C, with a light and dark period of 16/8 hr and a light intensity of 34 μmol/m^2^ s. For fresh weight determination, the shoot cultures were gently pressed on sterile filter papers to remove excess water and weighed. The developing shoot clusters were sub-cultured onto the same medium composition every four weeks for further shoot proliferation and elongation of shoots.

### Container closure

This experiment was carried out to test the influence of the type of container closure optimum for *in vitro* shoot cultures of *G. tenuifolia* . Glass bottles used as culture vessels were closed (sealed) with 4 dispensable papers (DP4) or with 2 layers of aluminum foil (AF2). In case of DP4, culture containers were additionally closed with a layer of parafilm which was removed after 2 weeks to facilitate a better gas exchange.

### Rooting of *in vitro* shoots

For induction of rooting, *in vitro* shoots were cultured in ½X MS basal medium supplemented with Indole-3-acetic acid (IAA) at 0.5, 1, 3 and 5 mg/L concentrations or Idole-3-butyric acid (IBA) at 0.05, 0.1, 0.5 and 1 mg/L concentrations. Sucrose (3%) and agar (0.9%) was added to all the media. Observations were recorded after 35 days of culture.

### Hardening and survival of tissue culture plants

Rooted plantlets were removed from the culture vessels, rinsed with water to remove the medium, and then transferred to plastic pots (9 cm diameter) containing a mixture of peat soil: perlite: vermiculite (1:1:1 v/v) in the greenhouse. The plants were watered once a day. Initially, a higher humidity was maintained by keeping the pots in a tray having water. Each pot was covered with a thin transparent polyethylene bag (sachet). After one week, one top corner of the sachet was cut. During the third week, a similar cut was made on the other side of the sachet. This sachet was completely removed in the fourth week. During the fifth week, pots were taken out of the tray. The data on survival of plants was recorded after five weeks of transfer to the greenhouse.

### HPLC analysis of tissue culture plants and commercial crude drug

#### Reagents, materials and conditions

HPLC-grade methanol was purchased from Merck (Germany). Pump (L-2130), auto injector (L-2200) and diode array detector system (L-2450) used were from Hitachi. Symmetry Water Column C_18_ (5 μm, 4.6 × 250 mm) and Milli Q water (Millipore, Milford, MA, USA) were used for all the analysis carried out at room temperature. The mobile phase for luteolin was a gradient eluting with acetonitrile/water (0.5% acetic acid) (from 20:80 to 100:0, by v/v) at 1 ml/min over 47 min. The eluent was monitored at 245 nm. While, mobile phase for oleanolic acid was a mixture of reagent acetonitrile and water (05% acetic acid) (90:10) at 1 ml/min over 15 min. The eluent was monitored at 210 nm.

#### Preparation of HPLC standard and samples

Luteolin (ChromaDex) and oleanolic acid (Fluka Analytical) standard samples were purchased from Sigma-Aldrich Co. LLC. . Standard solutions were prepared by dissolving 5 mg of each in 5 ml of Ethanol. Dissolved solutions (1.0 mg ml^−1^) were filtered through a 0.22 μm filter (Millipore, USA) and further diluted to the concentration of

1. For Luteolin: 1.0, 2.0, 4.0, 8.0 and 16 mg l^−1^.
2. For Oleanolic acid: 5.0, 10.0, 20.0, 40.0, 80.0 and 160.0 mg l^−1^.

Calibration curves for standards were established and high linearity (r2 > 0.999) was obtained for each calibration curve. Standard solution (10 μl) was used for HPLC injections. Calibration graphs were plotted based on linear regression analyses of the peak areas in response to concentrations of standards injected. The repeatability of the migration time and peak areas of Luteolin and oleanolic acid in the experiment were determined under the optimum conditions. Samples of *in vitro* shoots for the HPLC analysis were collected from the culture vessels and their fresh weights were recorded. The samples were then freeze-dried for 24 h and their dry weights were determined. Fraction (100 mg) of each dried sample was crushed into fine powder and dissolved in 10 ml of ethanol. It was ultra-sonicated for 10 min and the supernatant was collected after centrifugation (5000 rpm, 10 min). This process was repeated three times for each sample. After filtration, the combined ethanol extracts were evaporated to dryness with the help of a rotary evaporator. The residue was dissolved in 10 ml ethanol and filtered through a 0.22 μm (Millipore, USA) membrane before analysis.

#### Statistical analysis

Each treatment consisted of thirty replicates and each experiment was repeated three times. Data were statistically analyzed for least significant difference (LSD) using SAS 8.2 statistical software (SAS Institute Inc [2001]).

## Results and discussion

### Induction of multiple shoots

**Figure 1:**
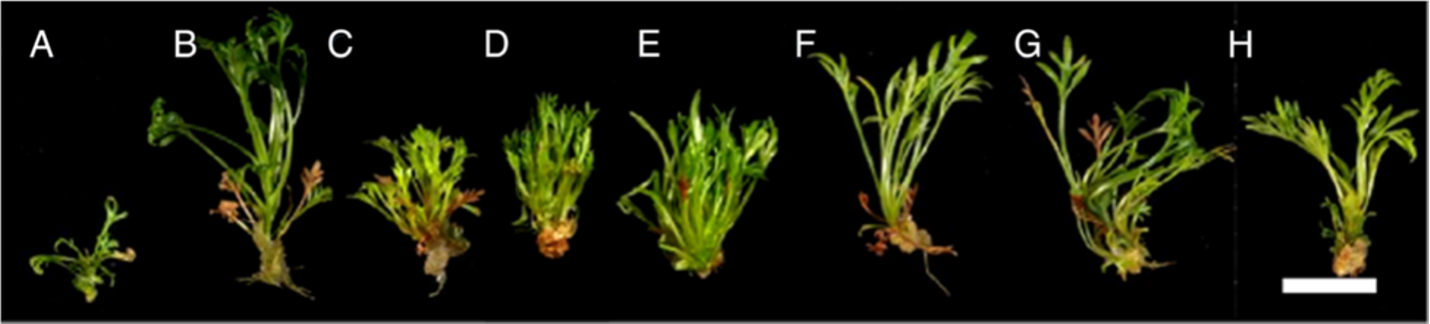
**Influence of BA and Kinetin on induction of multiple shoots in seedling-derived shoot tip of** ***G. tenuifolia*** **.** (**A**) ½X MS basal medium without PGRs; (**B**) 0.1 mg/L NAA; (**C**) 0.1 mg/L BA and 0.1 mg/L NAA; (**D**) 0.5 mg/L BA and 0.1 mg/L NAA; (**E**) 1 mg/L BA and 0.1 mg/L NAA; (**F**) 0.1 mg/L Kin and 0.1 mg/L NAA; (**G**) 0.5 mg/L Kin and 0.1 mg/L NAA; (**H**) 1 mg/L Kin and 0.1 mg/L NAA. (Bar = 1 cm)

Surface-sterilized seeds of *G. tenuifolia* inoculated on different basal media commenced gemination after 3 days of inoculation and a maximum 50% of seed germination at day 15 was recorded. The induction of multiple shoots in explants varied with cytokinin type and concentration (Table 1) (Figure 1). The response in medium supplemented with BA (1.0 mg/L) + NAA (0.1 mg/L) was higher compared to the medium with kinetin + NAA, or devoid of growth regulators. The maximum average number of shoots (7.4 ± 0.6) in 100% explants was obtained on ½X MS medium supplemented with BA (1.0 mg/L) + NAA (0.1 mg/L) and 3% sucrose after 35 days of inoculation. Also, this treatment resulted in the maximum fresh weight (894.9 ± 78.4) per explant. Multiple shoots developed directly from the lateral bud meristems. Kinetin and medium without plant growth regulators though supported elongation of shoots, but responses were lower compared to BA which induced a higher number of multiple shoots at all three concentrations (0.1, 0.5 and 1.0 mg/L) (Table 1).

**Table 1:** **Influence of BA and Kin on induction of multiple shoots in seedling-derived shoot tip explants of** ***G. tenuifolia***

Cytokinin*	NAA	No. of explants cultured	Shoot length (mm)**	Explants induced multiple Shoots (%)**	Average No. of shoots / explant**	Explants induced callus (%)**	Fresh weight/shoot (mg)**
(mg/L)	(mg/L)						
0	0	30	26.7 ± 2.1 cd	53.3 ± 9.1 bc	1.8 ± 0.3 c	0.0 ± 0.0 d	128.0 ± 16.7 d
0	0.1	30	40.8 ± 2.2 a	50.0 ± 9.1 bc	1.8 ± 0.2 c	56.7 ± 9.0 b	379.5 ± 33.4 c
BA 0.1	0.1	30	24.4 ± 1.3 d	93.3 ± 4.6 a	4.1 ± 0.4 b	33.3 ± 8.6 c	364.1 ± 43.6 c
BA 0.5	0.1	30	24.2 ± 1.4 d	100.0 ± 0.0 a	7.3 ± 0.6 a	13.3 ± 6.2 d	666.2 ± 68.4 b
BA 1.0	0.1	30	26.2 ± 1.7cd	100.0 ± 0.0 a	7.4 ± 0.6 a	95.0 ± 4.9 a	894.9 ± 78.4 a
Kin 0.1	0.1	30	40.1 ± 3.5 a	43.3 ± 9.0 c	1.8 ± 0.2 c	50.0 ± 9.1 bc	319.8 ± 44.2 c
Kin 0.5	0.1	30	32.8 ± 2.4 bc	70.0 ± 8.4 b	2.5 ± 0.3 c	80.0 ± 7.3 a	306.7 ± 32.6 c
Kin 1.0	0.1	30	34.6 ± 2.1 ab	56.7 ± 9.0 bc	2.3 ± 0.3 c	100.0 ± 0.0 a	253.0 ± 26.0cd

*Basal medium: ½X MS basal medium supplemented with 3% sucrose and 0.9% Difco Bacto-agar. Observations were recorded after 35 days of culture.

**Means followed by the same letter are not significantly different at 5% level by LSD (least significant difference) test.

Similar to the results in the present study, the differential effect of various concentrations of BAP on the induction of multiple shoots has earlier been reported for *Gossypium* (Agrawal et al. [1997]), *Salix* (Agrawal and Gebhardt [1994]), *Pisum* (Jackson and Hobbs [1990]) *Phaseolus* (McClean and Grafton [1989]) and *Glycine* (Cheng et al. [1980]). Akin to the present study, BAP was the most effective cytokinin in all these reports, indicating a particular cytokinin preference for multiple shoot induction in certain tissues.

### Container closure

**Figure 2:**
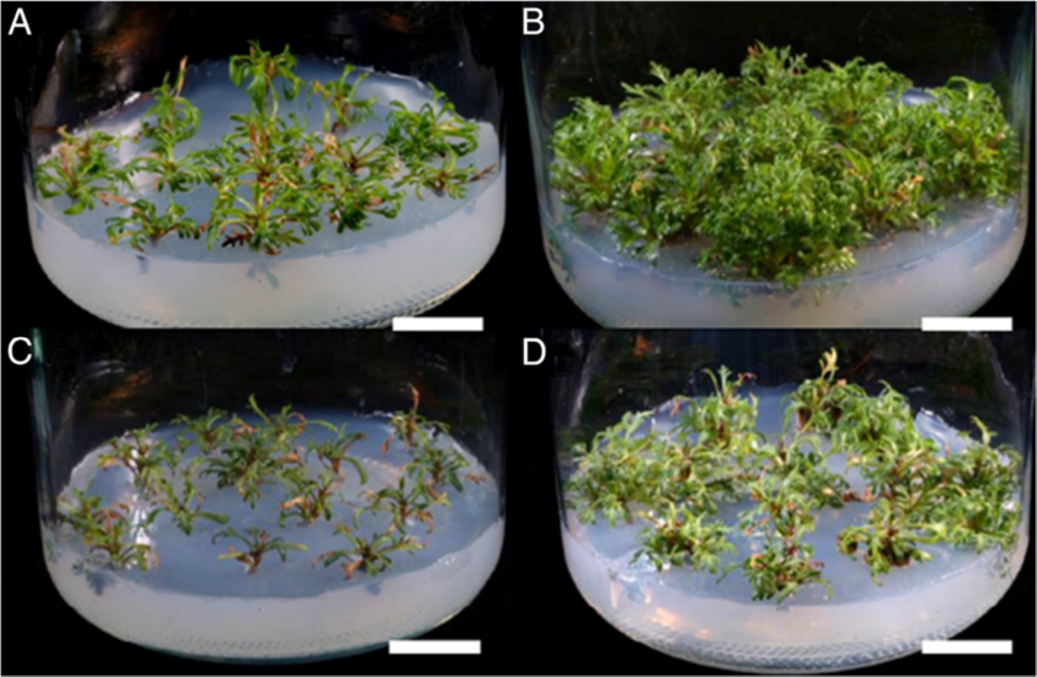
**Influence of container closure type on induction of multiple shoots in** ***G. tenuifolia*** **.** (**A**) Sealed with 2 layers of AF; ½X MS basal (**B**) Sealed with 2 layers of AF; BA 0.1 mg/L + 0.1 mg/L (**C**) Sealed with 4 layers of DPs; ½X MS basal; (**D**) Sealed with 4 layers of DPs; BA 0.5 mg/L + NAA 0.1 mg/L. (Bar = 2 cm).

Our experiment to find out the type of container closure optimum for *in vitro* shoot cultures of *G. tenuifolia* showed that the maximum average number of multiple shoots (9.7/ explant) and average fresh weight of each shoot (472.1 mg) was obtained when each culture container was closed with 2 layers of aluminum foil (AF). The container closure with 4 dispensable papers (DP4) resulted in a lower average number of shoots to 7.1/explant and fresh weight of 326.2 mg (Table 2) (Figure 2).

**Table 2:** **Influence of container closure type on induction of multiple shoots in** ***G. tenuifolia***

Ventilation closure*	Culture medium	Explants induced multiple shoots (%)***	Average No. of shoots / explant ***	Fresh weight / shoot (mg) ***
AF2	½ X MS basal	61.4 ± 6.2 b	2.3 ± 0.2 c	128.7 ± 6.8 c
AF2	NAA (0.1) + BA (0.1)	100.0 ± 0.0 a	9.7 ± 0.4 a	472.1 ± 15.2 a
DP4 **	½ X MS basal	68.6 ± 2.5 b	2.5 ± 0.1 c	73.5 ± 2.8 d
DP4	NAA (0.1) + BA (0.1)	100.0 ± 0.0 a	7.1 ± 0.5 b	326.2 ± 12.2 b

*Basal medium:½ X MS basal salts supplemented with 3% sucrose and 0.9% Difco Bacto-agar. Concentrations of PGRs in the parentheses represent mg/L values. Observations were recorded after 35 days of culture.

** AF2 = Culture container closed with 2 layers of Aluminum foil;

DP4 = Culture container sealed with 4 dispense papers. Culture containers were initially sealed with 4 dispense paper and parafilm layers. After 2 weeks, parafilm layer was removed to facilitate ventilation.

***Means followed by the same letter are not significantly different at 5% level by LSD (least significant difference) test.

To maintain the sterility of cultures, it is essential to close culture containers with some closure. Different types of container closures are commonly used. Some closures cause restriction of gaseous exchange between the container atmosphere and the outside environment (Buddendorf-Joosten and Woltering [1994]), which can result in poor aeration and hyperhydric condition of cultures. It has been reported that the type of closure affects gaseous exchange, availability of water, micronutrients, and balance of hormones in the culture container (Kataeva et al. [1991]; Lai et al. [2005]; Chen et al. [2006]; Tsay et al. [2006]). Also, growth rate and other physiological and morphological characteristics of plants developed under *in vitro* conditions can be influenced by the physical and chemical micro-environments of culture containers (Walker et al. [1988]). Different species show different requirement with respect to container closures. Hence, it is important to optimize a closure type in a micropropagation protocol of a particular plant species.

### Rooting of in vitro shoots

Between the two auxins tested, the response of IAA was found better compared to IBA for induction of rooting in *in vitro* shoots of *G. tenuifolia*. Half strength (½X) MS basal medium supplemented with 3.0 mg/L IAA induced an average number of 6.6 roots/shoot in 100% shoots (Table 3). The response of IBA in the medium was also comparable (6.3 roots/shoot), however, with IAA, the percentage of rooting was lower (83.3). Higher concentrations of both the auxins (>3.0 mg/L IAA and > 0.1 mg/L IBA) induced more average number of roots per shoot, however, shoots induced callus at the base which affected the survival rates of the rooted shoots in the greenhouse (Table 3). The promotory effect of a lower salt concentration of MS on *in vitro* rooting of shoots has been reported for *Gossypium* (Agrawal et al. [1997]), *Philodendron* spp. (Maene and Debergh [1985]). Different plant species respond differently to auxins for the induction of rooting. Some plant species, even do not require any auxin supplemental in the medium for rooting (Agrawal and Gebhardt [1994]), hence it is desirable to optimize the type and concentration of an auxin in a micropropagation protocol of a particular plant species.

**Table 3:** **Influence of IAA and IBA concentrations on** ***in vitro*** **rooting of shoots of** ***G. tenuifolia***

Auxin (mg/L) *	Concentration (mg/L)	No. of shoots rooted (%)**	Average No. of roots **	Callus formation	Plants survival in greenhouse (%)
IAA	0.5	53.3 ± 9.1 b	2.4 ± 0.4 cd	-	93.3
-	1.0	63.3 ± 8.8 b	3.3 ± 0.5 c	-	100.0
-	3.0	100.0 ± 0.0 a	6.6 ± 0.7 b	+	100.0
	5.0	93.3 ± 4.6 ab	9.5 ± 0.9 a	++	96.7
IBA	0.05	3.3 ± 3.4 d	0.1 ± 0.1 e	-	90.0
	0.1	16.7 ± 6.8 c	0.9 ± 0.3 de	-	96.7
	0.5	83.3 ± 6.8 ab	6.3 ± 0.7 b	+	96.7
	1.0	96.7 ± 3.3 ab	8.6 ± 0.7 a	+++	66.7

*Basal medium:½X MS basal medium supplemented with 3% sucrose and 0.9% Difco Bacto-agar. Observations were recorded after 35 days of culture.

**Means followed by the same letter are not significantly different at 5% level by LSD (least significant difference) test.

### Hardening and survival of tissue culture plants

**Figure 3:**
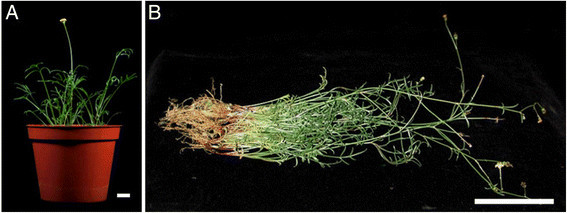
**(A) Tissue culture plants of** ***G. tenuifolia*** **successfully acclimatized, (B) Tissue culture plants in greenhouse (3½ month old).** Bar A = 1.3 cm;Bar B = 6 cm)

Hardening and 100% survival of tissue culture plants was achieved on the peat soil: perlite: vermiculite (1:1:1 v/v) mix in plastic pots kept in the greenhouse. Covering of plants with transparent sachets raised humidity levels, crucial for the survival of tissue culture plants of *G. tenuifolia*. Normal flowering and seed formation (Figure 3A, B) was observed in all the tissue culture raised plants after 3½ months of transfer to pots.

### HPLC analysis of secondary metabolites

HPLC analysis revealed the varying quantities of oleanolic acid and luteolin in *in vitro* shoots, tissue culture plants in the greenhouse, wild type and commercial crude drug materials (Table 4). The oleanolic acid and luteolin contents were found to be significantly higher (16.89 mg/g and 0.84 mg/g, respectively) in 3½-month old tissue culture raised plants in greenhouse compared to commercially available crude drug (6.51 mg/g, 0.13 mg/g, respectively). In comparison with the tissue culture plants in the greenhouse, the next best values of oleanolic acid and luteolin contents were recorded in wild type plants from Chimei and Wangan islands. There was only a marginal difference between the two wild type plant materials showing comparable quantities of both the active compounds. These compounds were present mostly in the aboveground parts of the plant. Only underground dried herbs of commercial crude drug showed oleanolic acid content, indicating probable mixing of aboveground parts with the underground parts or a wrong identification of the commercial crude drug. *In vitro* shoots growing under culture conditions on ½ strength MS basal medium devoid of any growth regulators also showed the presence of active compounds (3.29 mg/g of oleanolic acid and 0.47 mg/g of luteolin) indicating onset of biosynthetic pathways for production of these compounds at the culture stage itself. The lower quantities of the compounds in *in vitro* shoots in comparison to the tissue culture raised greenhouse plants may be due to differences in the maturity of the plant materials. Media supplemented with PGRs did not have significant influence on the contents of oleanolic acid and luteolin in *in vitro* shoots (Data not shown).

**Table 4:** **HPLC analysis for luteolin and oleanolic acid contents in** ***in vitro*** **raised, wild and commercially available plant materials of** ***Glossogyne tenuifolia***

Plant Samples	Source / Treatment	Oleanolic acid (mg/g of dw)*	Luteolin (mg/g of dw)*
Commercial crude drug	Dried herbs(Aboveground)	6.51	0.13
	Dried herbs(Underground)	1.07	none
Wild type	Chimei Island (Aboveground)	13.78	0.82
	Chimei Island (Underground)	none	none
	Wangan Island (aboveground)	14.58	0.72
	Wangan Island (Underground)	none	none
*In vitro* shoots	½X MS basal medium (−PGRs)	3.29	0.47
Tissue culture plants (3 month old) in greenhouse	Aboveground parts	16.89	0.84
	Underground parts	none	none

dw: Freeze-dried weight

Similar to the present study, significantly higher amounts of emodin and physcion contents were observed in *in vitro* propagated shoots and tissue culturplants of *Polygonum multiflorum* compared to the marketed crude drug (Lin et al. [2003]). Fairly high amounts of gentiopicroside and swertiamarin compounds were recorded in the aerial and underground parts of *Gentiana davidii* var. *formosana* compared to commercially available crude drug (Chueh et al. [2001]). Yet in another study on *Saussurea involucrata* in our laboratory, the highest Syringetin and Rutin contents were recorded in petioles of two months old *in vitro* plants compared to the commercially available crude drug (unpublished results). The possible reasons for the enhanced levels of active compounds in tissue culture plants could be, that the plants collected for the crude drugs are grown under natural conditions and the contents of the active compounds may vary depending on the growth conditions, place and time of plant collections. Whereas, the tissue culture plants are grown under controlled growth and environmental conditions receiving an optimum supply of nutrients and other favorable growth conditions. Based on the results of the present study, it is evident that under defined culture conditions, it is possible to produce plants with higher contents of oleanolic acid and luteolin contents in a shorter time span and throughout the year.

## Conclusions

In the present study, we have developed an *in vitro* propagation protocol for *G. tenuifolia* , an important medicinal plant native to Taiwan. HPLC analysis of tissue culture raised plants grown in the greenhouse have shown significantly higher levels of the active compounds compared to wild types and commercial crude drug, demonstrating the usefulness of the tissue culture technology. The results obtained in the present study have enormous significance, since so far there is no published report on micropropagation of this medicinally important plant species.

## Authors’ original submitted files for images

Below are the links to the authors’ original submitted files for images. Authors’ original file for figure 1 Authors’ original file for figure 2 Authors’ original file for figure 3

## Abbreviations

GT: *Glossogyne tenuifolia*:
MS: Murashige and Skoog ([1962]):
B_5_: Gamborg *et al*. ([1968]):
N_6_: Chu *et al*. ([1975]):
WPM: Loyd and McCown ([1981]) woody plant medium:
Kinetin: 6-furfurylamino purine:
BA: N6-benzyladenine:
NAA: α-naphthaleneacetic acid:
IAA: Indole-3-acetic acid:
IBA: Indole-3-butyric acid:
HPLC: High Performance Liquid Chromatography:
