# Supplementary figures and images for: In vitro propagation and analysis of secondary metabolites in Glossogyne tenuifolia (Hsiang-Ju) - a medicinal plant native to Taiwan
Source: Bot Stud. 2014 Jun 24;55:45. doi: 10.1186/s40529-014-0045-7 (PMC5432824; doi:10.1186/s40529-014-0045-7)

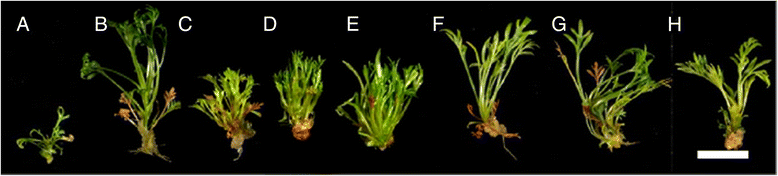

Supplement: Supplementary file 1 — Authors’ original file for figure 1 [file 40529_2014_45_MOESM1_ESM.gif]

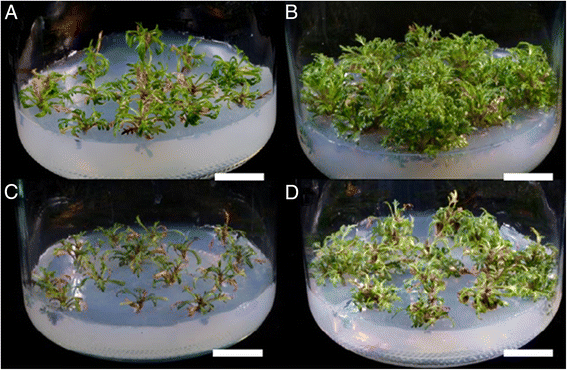

Supplement: Supplementary file 2 — Authors’ original file for figure 2 [file 40529_2014_45_MOESM2_ESM.gif]

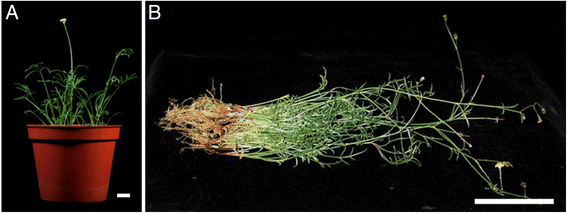

Supplement: Supplementary file 3 — Authors’ original file for figure 3 [file 40529_2014_45_MOESM3_ESM.gif]
